# Nutrient Intake Amongst Rural Adolescent Girls of Wardha

**DOI:** 10.4103/0970-0218.69264

**Published:** 2010-07

**Authors:** CH Maliye, PR Deshmukh, SS Gupta, S Kaur, AM Mehendale, BS Garg

**Affiliations:** Dr. Sushila Nayar School of Public Health, Mahatma Gandhi Institute of Medical Sciences, Sewagram, India

**Keywords:** BMI, calorie, dietary recall, iron, protein

## Abstract

**Objective:**

To assess the nutrient intake of rural adolescent girls.

**Materials and Methods:**

The cross-sectional study was carried in four adopted villages of the Department of Community Medicine, M.G.I.M.S., Sewagram. A household survey was carried out in the villages. A list of all the adolescent girls in the age group of 10-19 years was prepared by enumeration through house-to-house visit. All adolescent girls were included in the study. A pre-designed and pre-tested questionnaire was used to collect data on socio-demographic variables and anthropometric variables. A 24 h recall method was used to assess nutrient intake. Data generated was entered and analyzed using epi_info 2000. Nutrient intake was compared with ICMR Recommended Dietary Allowances. Nutritional status was assessed by BMI for age.

**Results:**

The mean height of the adolescent girls was 142.9 cm. Overall, 57% of the adolescents were thin (BMI for age <5^th^ percentile for CDC 2000 reference) and 43% of the adolescents were normal (BMI for age between 5^th^ – 85^th^ percentile for CDC 2000 reference). The average energy intake, which was 1239.6±176.4 kcal/day, was deficient of RDA by 39%. The average protein intake was 39.5±7 gm/day. It was deficient by 36% and the average iron intake, which was 13.2±2.5 mg/day, was deficient by 48%.

**Conclusions:**

The findings reiterate the dietary deficiency among adolescent girls which adversely affects the nutritional status. If the poor nutritional status is not corrected promptly before they become pregnant, it adversely affects the reproductive outcome. If we have to meet out the goals of Reproductive and Child Health Program, intervention strategies to improve the dietary intake of adolescent girls are needed so that their requirements of energy, protein, vitamins and minerals are met.

## Introduction

There are nearly one billion adolescents in the world accounting for 20-25% of the total population in the developing countries. This particular group of population is likely to increase rapidly in the next 30 years due to population momentum effect.(1) Owing to sudden and special growth taking place in this phase, the nutritional requirements also increase tremendously compared to preceding years of growth. During this phase, diet should provide not only sufficient calories but also essential elements and nutrients such as protein, vitamins and minerals required for growth.

Nutrition is an input to the foundation for health and development. Better nutrition is a prime entry point to ending poverty and a milestone to achieving better quality of life. Freedom from malnutrition is a basic human right and their alleviation is a fundamental prerequisite for human and national development. Malnutrition is associated with significant morbidity, mortality and economic costs in developing countries.(2) It also affects the reproductive outcome of the mother. Interventions which targeted pregnant mothers failed to improve the reproductive outcomes and there is an urgent need to improve the nutritional status before a woman becomes pregnant.(2) To design appropriate strategy to tackle the poor nutrition among adolescent girls and eventual morbidity and mortality, it is essential to study the dietary pattern. Hence, the present study was undertaken to know the nutrient intake among adolescent girls of rural Wardha.

## Materials and Methods

A cross-sectional study was carried out in the four adopted villages of the Department of Community Medicine, Mahatma Gandhi Institute of Medical Sciences, Sewagram, Wardha. A household survey was carried in all the four villages to enumerate unmarried adolescent girls in the age group of 10-19 years. All the adolescent girls were included in the study. A predesigned and pre-tested questionnaire was used to collect data on socio-demographic and anthropometric variables. A 24 h recall method was used to assess nutrient intake. The nutrient intake was calculated using tables of nutritive value of Indian foods.(3) Data generated were entered and analyzed using epi info 2000. The CDC 2000 reference was used to assess the nutritional status.(2) The Chi-square test was used for testing statistical significance. The level of significance was taken at *P* value <0.05.

## Results

All the 430 unmarried adolescent girls enumerated, participated in the study. Overall, 57% of the adolescent girls were thin and 43% were normal. None of them were overweight or obese. The prevalence of thinness was significantly higher 67.6% in early adolescence than in late adolescence 55.4%.

Majority of the adolescent girls (82.5%) had calorie intake less than 1400 kcal. 7.5% girls had calorie intake less than 1000 kcal. The average energy intake was 1239.6±176.4 kcal/day. The calorie intake of adolescent girls was less than the recommended dietary allowances for their age. The average calorie intake was deficient by 39%. The average protein intake was 39.5±7 gm/day which was deficient by 36%. The average iron intake was 13.2±2.5 mg/day and was deficient by 48% [Table 1].

**Table 1:** Nutrient intake of adolescent girls

Age (years)		Number Nutritional status** (BMI for age)		Calorie (kcal/day)			Proteins			Iron		
		<5^th^	5^th^-85^th^	RDA	Mean intake±SD	%Deficit*	RDA	Mean intake±SD	%Deficit*	RDA	Mean intake±SD	%Deficit*
10	36	25 (69.4)	11 (30.6)	1970	1130.8±130.0	42.6	57	35.8±6.1	37.2	19	11.52±2.1	39.3
11	39	30 (76.9)	9 (23.1)	1970	1177.1±198.9	40.2	57	38.5±7.3	32.4	19	12.85±2.7	32.3
12	50	34 (68.0)	16 (32.0)	1970	1208.0±163.3	38.7	57	39.2±6.6	31.3	19	12.96±2.4	31.7
13	52	34 (65.4)	18 (34.6)	2060	1224.1±149.6	40.5	65	38.1±5.4	41.3	28	12.81±1.9	54.2
14	41	24 (58.5)	17 (41.5)	2060	1244.5±173.6	39.6	65	38.8±6.9	40.1	28	13.39±2.3	52.1
15	49	24 (49.0)	25 (51.0)	2060	1282.1±169.2	37.8	65	40.9±7.0	36.9	28	13.90±2.5	55.3
16	47	23 (48.9)	24 (51.1)	2060	1243.6±159.5	39.6	63	39.4±5.9	37.4	30	13.24±2.2	55.8
17	49	23 (46.9)	26 (53.1)	2060	1322.6±183.4	35.8	63	42.3±7.8	32.8	30	13.98±2.7	53.4
18	34	17 (50.0)	17 (50.0)	2060	1260.8±195.4	38.8	63	40.4±8.4	35.8	30	13.68±2.6	54.4
19	33	11 (33.3)	22 (66.7)	2060	1284.1±179.4	37.6	63	40.6±7.2	35.5	30	13.97±2.4	53.4

Total	430	245 (57.0)	185 (43.0)	-	1239.6±176.4	39.1	-	39.5±7.0	36.1	-	13.2±2.5	48.2

* Percentage deficit in recommended intake;

** None of the participant had BMI for age >85^th^ percentile.

## Discussion

Poor nutritional status during adolescence is an important determinant of health outcomes. Short stature in adolescents resulting from chronic undernutrition is associated with reduced lean body mass and deficiencies in muscular strength and working capacity. In the present study, 57% of the adolescents were thin while 43% were normal. The high prevalence of thinness is reported from the developing world. National Nutrition Monitoring Bureau(4) also showed that the height, weight and growth rates of adolescents of low income groups were about 70-80% of those of well-to-do adolescents. Choudhary *et al*(5) reported that 68.52% of the adolescents had BMI less than 18.5 in rural area of Varanasi. In the present study, thinness was significantly higher in early adolescence (67.64%) than in late adolescence (55.42%). Deshmukh *et al*(6) reported that majority (53.8%) of the adolescents were thin, only 2.2% were overweight while 44.0% were normal. Medhi *et al*(7) reported that 41.3% of the adolescent girls were thin.

In the present study, the average energy intake was 1239.6±176.4 kcal/day and the calorie intake was deficient by 39%. Chaturvedi *et al*(8) reported that the calorie intake was deficient by 36%, 34% and 26% in the age group 10-12 years, 13-15 years and 16-18 years respectively. Yadav and Singh(9) reported that the calorie deficiency among adolescents was 29%.

The average protein intake was 39.5±7 gm/day and the protein intake was deficient by 36%. Chaturvedi *et al*(8) reported that in the age group 10-12 years, 13-15 years and 16-18 years, the protein deficit was 29%, 32% and 23% respectively. Yadav and Singh(9) reported that the magnitude of stunting was 60% among the adolescents.

The average iron intake was 13.2±2.5 mg/day and was deficient by 48.2%. Butley(10) found that the mean iron intake was 7±3.1 mg in the age group of 14-16 years in low socio-economic status, while in upper socioeconomic status, it was 18.5±5.2 mg. She also observed that in the age group of 17-18 years, the mean iron intake was 10.1±3.1 mg in lower socio-economic status, and in upper socio-economic status, it was 24.13.7 mg. Earlier diet surveys in adolescent population have also shown that the diets are inadequate in all nutrients including iron, proteins, calcium and calories.(13–15) Similar findings were also reported by Reddy(11) and Vasanthi *et al*.(12)

The findings reiterate the dietary deficiency among adolescent girls which adversely affects the nutritional status. If the poor nutritional status is not corrected promptly before they become pregnant, it will adversely affect the reproductive outcome. If we have to meet out the goals of Reproductive and Child Health Program, intervention strategies to improve the dietary intake of adolescent girls are needed so that their requirements of energy, protein, vitamins and minerals are met.
